# The association between specific cognitive domains function and gait performance or postural control in post-stroke participants: a cross-sectional study

**DOI:** 10.3389/fneur.2026.1679157

**Published:** 2026-01-20

**Authors:** Yana Wang, Lu Wang, Junnan Zhou, Meikui Deng, Yaqin Qiao, Jifeng Rong

**Affiliations:** 1 The Center of Rehabilitation Therapy, The First Rehabilitation Hospital of Shanghai, Shanghai, China; 2 School of Exercise and Health, Shanghai University of Sport, Shanghai, China; 3 Department of Rehabilitation Medicine, Shanghai Second People’s Hospital, Shanghai, China

**Keywords:** cognitive function, executive function, gait performance, postural control, stroke

## Abstract

**Background:**

Stroke survivors frequently experience cognitive dysfunction, impaired postural control, and gait impairments, significantly impacting physical abilities and hinders their ability to live independently. Although cognitive impairment exacerbates motor deficits, existing research primarily examines global cognition or executive function, with limited focus on domain-specific cognitive association. This study aims to investigate the distinct relationships between specific cognitive domains and both gait performance and postural control in stroke survivors.

**Methods:**

Thirty-three acute and subacute stroke participants (mean age 63.94 ± 10.3 years, 60.6% male, mean post-stroke duration 2.97 ± 2.86 months) underwent standardized assessments: Executive function (Shape Trail Test, STT), attention (Symbol Digit Modalities Test, SDMT), Visuospatial ability and memory (Rey-Osterrieth Complex Figure Test, ROCFT), gait (10-Meter Walk Test, 10MWT, Timed Up and Go Test, TUG), and postural control (NeuroCom Balance System parameters: Movement Velocity (MVL), Max Excursions (MXE), and Directional Control (DCL)). Domain-specific associations between cognitive functions and gait performance or postural control were analyzed using multiple linear regression, with adjustment for age, sex, time since onset, stroke type, and hemiparetic side.

**Results:**

The results revealed that better executive function significantly predicted faster MVL and greater MXE during postural control (*p* < 0.01). In contrast, superior visual memory was associated with slower MVL (*p* = 0.027). However, no cognitive domains significantly predicted performance on the 10MWT or TUG.

**Conclusion:**

Impairments in specific cognitive domains differentially correlate with gait and postural control, underscoring the need for integrated cognitive-motor rehabilitation in acute and subacute stroke.

## Introduction

Stroke is a major global health challenge, representing a leading cause of long-term adult disability worldwide. The incidence of stroke continues to rise, placing an increasing burden on healthcare systems and society (1). In the aftermath of a stroke, survivors often present with a complex interplay of walking disabilities, postural control difficulties, and cognitive disorders. Specifically, up to 60% of survivors experience cognitive decline in the first year after stroke, with executive dysfunction, attention deficits, and memory problems being the most common (2); 54 to 80% of participants suffer from persistent gait disturbance in the early recovery phase, which severely limits their functional mobility (3). Furthermore, a majority of stroke participants suffer from postural control dysfunctions, manifesting as static and dynamic instability, significantly increasing the risk of falls and secondary injuries (4). These combined deficits severely affect the quality of life and limit social participation among stroke participants (5, 6).

A core consensus in the current field of post-stroke rehabilitation is that safe, stable, and coordinated motor function is not regulated solely by the motor system (7, 8). Cognitive processes, primarily executive function and attentional control, are crucial for walking performance and postural stability, as these activities demand continuous cognitive resource allocation for tasks such as obstacle negotiation, balance correction, and motor planning (9, 10). Based on this understanding, a growing body of research has begun to explore the associations between global cognitive function, gait, and postural control in stroke participants (11–15). Study by Yu et al. revealed that complex mobility tasks such as turning and sit-to-stand transfers demand more cognitive resources compared to straight walking, with overall cognitive function playing a key role (13). Furthermore, several studies have focused on executive function, revealing its correlation with both walking ability and postural control (10–14). Despite these valuable insights, a critical limitation persists. Although stroke typically induces multidomain cognitive dysfunction, the existing literature has predominantly focused on executive function or global cognitive scores (16, 17). The unique, distinct contributions of other compromised domains, such as attention, visuospatial construction, and visual memory, to specific aspects of walking ability and postural control remain inadequately delineated. A more granular, domain-specific understanding is essential to move beyond phenomenological description and toward mechanistic explanation.

This exploratory study was specifically designed to address this identified gap and hypothesize that specific cognitive domains will demonstrate dissociable associations with distinct motor functions. By elucidating these specific relationships, our findings aim to provide a preliminary foundation for developing targeted, cognitive-domain-specific rehabilitation strategies, ultimately aiming to improve functional outcomes and quality of life after stroke.

## Materials and methods

### Study participants

This preliminary cross-sectional study was conducted at the First Rehabilitation Hospital of Shanghai, China. Between December 2023 and December 2024, 33 stroke survivors were consecutively enrolled. The sample size was estimated *a priori* using G*Power 3.1 software. Based on the primary outcome and referencing a medium effect size (Cohen’s f^2^ = 0.3) from a comparable study on cognition-posture control in stroke by Yu et al. (13), the required sample size was calculated as 30 participants, given an alpha level of 0.05 and a power (1-*β*) of 0.8. The final enrollment of 33 participants therefore met the basic statistical power requirement. All participants satisfied the predefined eligibility criteria.

The inclusion criteria were as follows: (1) Confirmed diagnosis of a first-time stroke, (2) Presence of unilateral hemiplegia, (3) Age between 40 to 80 years, (4) Ability to walk 10 meters without assistance, (5) Stroke occurrence within the past 6 months. The exclusion criteria were as follows: (1) Other cerebrovascular diseases, (2) Sensory aphasia, (3) Traumatic hemorrhage, (4) Sensory disorders, (5) Concurrent bone and joint system diseases.

## Methods

### Cognitive assessments

#### Shape Trail Test (STT)

STT is administered to assess executive function. In this test, participants were presented with a series of circles and squares containing numbers and were required to connect them in alternating sequence (circle-square-circle…) as quickly as possible. The total time taken to complete the task was recorded, with longer completion times indicating greater impairment in executive functions. The STT has demonstrated good test–retest reliability and validity in assessing executive function among stroke participants (18, 19).

#### Symbol digit modalities test (SDMT)

The SDMT is a tool for assessing attention and information processing speed. The test includes nine symbol-number pairs, which are randomly repeated 120 times. Participants were instructed to match each symbol to its corresponding number within 90 s. The number of correct matches was recorded, with lower scores indicating greater attention impairment. The SDMT is a widely used measure with established high reliability and validity in neurological populations, including stroke survivors (20, 21).

#### Rey-Osterrieth complex figure test (ROCFT)

The ROCFT is a tool for evaluating visuospatial construction and visual memory. The test consists of two parts. Participants were first asked to copy the figure, which evaluates visuospatial construction abilities, and 20 min later, they were asked to reproduce it again, assessing long-term visual memory. To obtain a more quantitative measure of drawing accuracy, this study uses the ROCFT with the Osterrieth scoring criteria to diagnose cognitive impairment, with lower scores indicating more severe impairment. The ROCFT demonstrates excellent reliability and is a well-validated tool for assessing visuospatial function in stroke populations (22, 23).

#### Walking ability and postural control assessments

All motor and postural control assessments were conducted in a dedicated, well-lit indoor rehabilitation hall to ensure safety and environmental consistency. Participants were assessed wearing their own regular walking or rehabilitation shoes, and conditions were kept consistent across trials. Prior to each test, participants received a demonstration and were allowed one practice trial to ensure comprehension. Data were collected only during subsequent formal trials.

#### 10 meter walk test (10MWT)

The 10MWT is used to assess walking speed. Participants were instructed to walk at a comfortable, self-selected pace along a 14-meter walkway, which included 2-meter acceleration and 2-meter deceleration zones at each end of the timed 10-meter central section. The time taken to traverse the central 10 meters was recorded to calculate gait speed. Higher gait speeds indicate better walking performance. The 10MWT is a highly reliable and valid measure of gait speed in the stroke population (24).

#### The timed up and go test (TUG)

The TUG evaluates mobility ability and can be used as an indicator of fall risk. Participants were timed as they stood up from a standard armchair, walked 3 meters at a safe and comfortable pace, turned around, walked back to the chair, and sat down again. Shorter completion times reflect better functional mobility and lower fall risk. The TUG has shown excellent reliability and is a valid functional mobility measure for individuals with stroke (25).

### NeuroCom Balance System

Posture control is evaluated using the NeuroCom Balance System (NeuroCom Balance Manager, USA) with the Limits of Stability test. During the assessment, participants stood on the force plate with their eyes open, feet shoulder-width apart, and arms relaxed at their sides. They were instructed to shift their center of mass toward eight sequentially presented visual targets: front, right-front, right, right-back, back, left-back, left, and left-front, following on-screen cues. Prior to formal testing, each participant completed two full practice trials to ensure comprehension of the instructions; practice data were excluded from the final analysis. The test generates five key indicators: Movement Velocity (MVL), Max Excursions (MXE), and Directional Control (DCL). A higher MVL indicates more rapid balance adjustments. MXE is expressed as percentages relative to the body’s stability limits, with higher values indicating better stability. And lower DCL suggest greater deviations from straight-line motion.

### Statistical analysis

All statistical analyses were performed using IBM SPSS Statistics version 25.0. A two-sided *p*-value of < 0.05 was considered statistically significant. Univariate analyses were first conducted to identify potential covariates associated with motor outcomes. The associations between categorical variables (sex, stroke type, hemiplegic side) and motor outcomes were assessed using independent samples t-tests. Continuous variables (age, time since stroke onset) were assessed using simple linear regression models with the motor score as the dependent variable. Based on previous literature and clinical relevance, all variables with a *p*-value < 0.1 in the univariate analysis, as well as those deemed *a priori* to be important potential confounders, were selected as covariates for the subsequent multivariate models. To investigate the independent predictive role of cognitive function on motor recovery, a series of multiple linear regression models were constructed using the enter method. Specifically, for each motor outcome (MVL, MXE, DCL, 10MWT, TUG), a separate regression model was built with the motor score as the dependent variable. In each model, the primary independent variable was cognitive measure (STT, SDMT, ROCFT-Copy, ROCFT-Delayed Recall), adjusted for all the covariates identified in the previous step. The results are presented as unstandardized regression coefficients (B), standardized coefficients (Beta) and *p*-values. Multicollinearity was assessed for all models, and the variance inflation factor (VIF) was confirmed to be below 10 for all independent variables, indicating acceptable levels of multicollinearity. The assumptions of linear regression, including linearity, independence of errors, and normality and homoscedasticity of residuals, were examined and deemed satisfactory.

## Results

We recruited 33 individuals (20 males, 13 females; mean age: 63.94 ± 10.3 years) at a subacute stage post-stroke (2.97 ± 2.86 months). The cohort, comprising both ischemic and hemorrhagic strokes (25/8), presented with global cognitive impairment (MoCA: 23.42 ± 3.45). The detailed results are summarized in Table 1.

**Table 1:** Characteristics of the participants.

Characteristics	Mean ± SD (*n* = 33)
Age (years)	63.94 ± 10.3
Hemiplegic limb (right/left)	15/18
Onset time (months)	2.97 ± 2.86
Sex (male/female)	20/13
Type of stroke (Inf/Hem)	25/8
STT	198.82 ± 86.56
SDMT	25.06 ± 9.48
ROCFT-Copy	25.85 ± 8.55
ROCFT-Delayed Recall	9.68 ± 7.50
MVL (deg/s)	2.60 ± 1.06
MXE (%)	60.74 ± 14.54
DCL (%)	58.50 ± 11.26
10MWT (s/10 m)	19.03 ± 11.44
TUG(s)	21.27 ± 10.56

STT, shape trail test; SDMT, symbol digit modalities test; ROCFT-Copy, Rey-Osterrieth complex figure test - copy; ROCFT-Delayed Recall, Rey-Osterrieth complex figure test - delayed recall; MVL, movement velocity; MXE, max excursions; DCL, directional control; 10MWT, 10 meter walk test; TUG, the timed up and go test.

### Regression model for MVL

Following variable selection, four cognitive indicators were included in the model. After controlling for covariates, the regression model for MVL was statistically significant overall (*F* = 5.381, *p* = 0.002) and accounted for 43.5% of the variance (R^2^ = 0.435). STT (B = −0.01, *p* < 0.001) and ROCFT-Delayed Recall (B = −0.07, *p* = 0.027) were significantly associated with MVL. In contrast, the SDMT and the ROCFT-Copy did not reach statistical significance. Results of this model indicated that better executive function and poorer visual memory were independently associated with faster movement velocity (Table 2).

**Table 2:** Multiple linear regression analysis for MVL.

Model		B	SE	Beta	*t*	*p*	VIF
Constant		6.046	1.217		4.968	<0.001	
Independent variable	STT	−0.01	0.003	−0.852	−4.082	**<0.001**	2.156
	SDMT	−0.042	0.024	−0.373	−1.769	0.088	2.206
	ROCFT-Copy	0.014	0.024	0.111	0.583	0.565	1.792
	ROCFT-Delayed Recall	−0.07	0.03	−0.495	−2.332	**0.027**	2.230
R^2^		0.435					
*F*		5.381					
*p*		**0.002**					

STT, shape trail test; SDMT, symbol digit modalities test; ROCFT-Copy, Rey-Osterrieth complex figure test - copy; ROCFT-Delayed Recall, Rey-Osterrieth complex figure test - delayed recall; B, unstandardized regression coefficient; SE, standard error; VIF, variance inflation factor. Significant *p*-values (< 0.05) are presented in bold.

### Regression model for MXE

Following variable selection, four cognitive indicators and the hemiplegic side were included in the regression model. After controlling for covariates, the regression model for MXE was statistically significant overall (*F* = 4.072, *p* = 0.007) and explained 43.0% of the variance (R^2^ = 0.430). STT (B = −0.106, p = 0.007) was the sole cognitive variable showing a significant association with MXE, suggesting that better executive function is significantly associated with higher motor stability. The other cognitive metrics did not reach statistical significance (Table 3).

**Table 3:** Multiple linear regression analysis for MXE.

Model		B	SE	Beta	*t*	*p*	VIF
Constant		92.094	17.065		5.397	<0.001	
Independent variable	STT	−0.106	0.036	−0.631	−2.944	**0.007**	2.174
	SDMT	−0.496	0.332	−0.323	−1.492	0.147	2.223
	ROCFT-Copy	−0.256	0.335	−0.150	−0.763	0.452	1.839
	ROCFT-Delayed Recall	0.338	0.421	0.174	0.804	0.429	2.233
Control variable	Hemiplegic limb (left)	10.008	4.417	0.348	2.266	0.032	1.118
	Hemiplegic limb (right)	0					
R^2^		0.430					
*F*		4.072					
*p*		**0.007**					

STT, shape trail test; SDMT, symbol digit modalities test; ROCFT-Copy, Rey-Osterrieth complex figure test - copy; ROCFT-Delayed Recall, Rey-Osterrieth complex figure test - delayed recall; B, unstandardized regression coefficient; SE, standard error; VIF, variance inflation factor. Significant *p*-values (< 0.05) are presented in bold.

### Regression model for DCL

Following variable selection, four cognitive indicators and the hemiplegic side were included in the regression model. After controlling for covariates, the regression model for DCL was statistically significant overall (*F* = 3.785, *p* = 0.010) and accounted for 41.2% of the variance (R^2^ = 0.412). However, none of the cognitive predictors in the model reached the level of statistical significance (all *p* > 0.05). Although visual memory (ROCFT-Delayed Recall) exhibited a relatively large positive coefficient (B = 0.565, *p* = 0.100), suggesting a possible association between better visual memory and greater movement deviation (higher DCL values), this only exhibited a trend (Table 4).

**Table 4:** Multiple linear regression analysis for DCL.

Model		B	SE	Beta	*t*	*p*	VIF
Constant		58.121	13.427		4.329	<0.001	
Independent variable	STT	−0.033	0.028	−0.256	−1.178	0.249	2.174
	SDMT	−0.041	0.261	−0.034	−0.157	0.877	2.223
	ROCFT-Copy	0.023	0.264	−0.018	−0.088	0.931	1.839
	ROCFT-Delayed Recall	0.565	0.331	0.376	1.706	0.100	2.233
Control variable	Hemiplegic limb (left)	5.804	3.475	0.261	1.670	0.106	1.118
	Hemiplegic limb (right)	0					
R^2^		0.412					
*F*		3.785					
*p*		**0.010**					

STT, shape trail test; SDMT, symbol digit modalities test; ROCFT-Copy, Rey-Osterrieth complex figure test - copy; ROCFT-Delayed Recall, Rey-Osterrieth complex figure test - delayed recall; B, unstandardized regression coefficient; SE, standard error; VIF, variance inflation factor. Significant *p*-values (< 0.05) are presented in bold.

### Regression models for 10MWT and TUG

Following variable selection, both regression models incorporated the same set of four cognitive indicators. After adjusting for covariates, neither of the regression models for the 10MWT (R^2^ = 0.143, *F* = 1.165, *p* = 0.348) nor the TUG (R^2^ = 0.220, *F* = 1.977, *p* = 0.125) was statistically significant. This indicates that the cognitive variables examined were not significantly associated with participants’ performance in these two functional walking tasks (Tables 5, 6).

**Table 5:** Multiple linear regression analysis for 10MWT.

Model		B	SE	Beta	*t*	*p*	VIF
Constant		20.064	16.145		1.243	0.224	
Independent variable	STT	0.029	0.034	0.217	0.843	0.406	2.156
	SDMT	−0.203	0.314	−0.168	−0.648	0.522	2.206
	ROCFT-Copy	0.059	0.314	−0.044	−0.187	0.853	1.792
	ROCFT-Delayed Recall	−0.012	0.399	−0.008	−0.030	0.976	2.233
R^2^		0.143					
*F*		1.165					
*p*		0.348					

STT, shape trail test; SDMT, symbol digit modalities test; ROCFT-Copy, Rey-Osterrieth complex figure test - copy; ROCFT-Delayed Recall, Rey-Osterrieth complex figure test - delayed recall; B, unstandardized regression coefficient; SE, standard error; VIF, variance inflation factor.

**Table 6:** Multiple linear regression analysis for TUG.

Model		B	SE	Beta	*t*	*p*	VIF
Constant		23.983	14.211		1.688	0.103	
Independent variable	STT	0.031	0.030	0.251	1.026	0.314	2.156
	SDMT	−0.242	0.276	−0.217	−0.876	0.389	2.206
	ROCFT-Copy	0.107	0.276	−0.086	−0.386	0.702	1.792
	ROCFT-Delayed Recall	0.000	0.351	0.000	−0.001	0.999	2.233
R^2^		0.220					
*F*		1.977					
*p*		0.125					

STT, shape trail test; SDMT, symbol digit modalities test; ROCFT-Copy, Rey-Osterrieth complex figure test - copy; ROCFT-Delayed Recall, Rey-Osterrieth complex figure test - delayed recall; B, unstandardized regression coefficient; SE, standard error; VIF, variance inflation factor.

## Discussion

Using multiple linear regression, this exploratory study examined the associations between four cognitive domains for walking ability and postural control in stroke participants. The preliminary results indicate that executive function serves as a core cognitive correlate of postural control, with better performance associated with increased movement velocity and maximum excursion. In contrast, visual memory demonstrated an inverse association, where superior performance were related with slower movement speed. No cognitive variables significantly predicted functional walking performance. These findings preliminarily suggest that during the acute and subacute phases post-stroke, the association of specific cognitive domains with motor function is task-dependent, being more evident in posture control tasks that impose higher cognitive demands than in single-task gait paradigms.

This study supports a close relationship between executive function and the optimization of MVL and MXE during postural control, consistent with its established role in motor control. Executive dysfunction is associated with impaired postural control, and cognitive flexibility—essential for adapting to environmental changes—has been linked to post-stroke balance recovery (13, 15). This association may be supported by the ability to rapidly inhibit automatic responses and switch strategies in dynamic settings, a process relevant to both MVL and MXE (26, 27). The shared neural substrate for this cognitive-motor relationship likely involves the frontal–cerebellar circuit (28–30). In line with this, integrity of the frontal–pontine–cerebellar pathway correlates with both executive function and dynamic balance in stroke participants (31). Within this framework, MVL may index this circuit’s efficiency in motor execution, while MXE may indicate its capacity for fine movement control. Collectively, these observations suggest that executive function is a marker of frontal–cerebellar circuit integrity, which facilitates the real-time adjustment between speed and stability during postural tasks.

A significant negative correlation was observed between visual memory and MVL during postural control. This finding contrasts with the generally positive associations reported between cognitive function and postural performance. Visual–spatial memory depends on constructing and maintaining detailed spatial mental models via the prefrontal-parietal network, a process requiring substantial central cognitive resources (32, 33). It is plausible that during posture tasks with concurrent spatial-cognitive demands, enhanced internal modeling may compete for resources necessary for real-time sensorimotor integration (34), potentially delaying movement initiation and resulting in reduced MVL. The absence of a significant association with maximum excursion further indicates that this speed reduction likely reflects cognitive modulation rather than a speed-accuracy trade-off. A faster MVL may instead signify shallower spatial processing or simplified cognitive resource allocation (35). These results underscore that the association between cognitive function and motor performance depends critically on the degree of shared cognitive resource demand. In clinical rehabilitation, individual cognitive profiles should be considered, and tasks could be adapted to optimize cognitive-motor synergy, rather than focusing solely on increasing speed.

Although the regression model for direction control was significant, no individual cognitive variable reached significance, a finding that aligns with prior research. For instance, Cui et al. observed that direction control is less sensitive to cognitive status than other postural metrics in older adults (36), a pattern replicated here in a stroke population. The regulation of movement trajectory straightness may rely more heavily on sensorimotor integration via cerebellar–brainstem pathways and real-time proprioceptive feedback than on the higher-order cognitive domains assessed.

Similarly, cognitive variables were not significantly associated with performance in functional walking tasks (10MWT, TUG). This differs from some previous reports (13, 15). A discrepancy potentially attributable to methodological differences such as variations in control for confounders. Moreover, single-task walking performance is primarily associated with physiological factors like lower limb strength (37). In neurological populations, cognitive associations with gait typically become evident only under dual-task conditions that impose explicit cognitive load (38). Although the TUG inherently involves executive demands, its single-task version might be insufficiently sensitive to detect cognitive effects when basic physical function varies substantially across individuals. Collectively, these observations imply that in stroke, single-task walking performance may be closely linked to physiological capacity, which could overshadow the contribution of specific cognitive domains.

Based on these exploratory findings, preliminary considerations for stroke rehabilitation can be suggested, with the clear understanding that they require validation before clinical application. In assessment, executive function could serve as a potential indicator of postural control risk; screening with the Shape Trail Test might aid in earlier fall risk identification. Regarding intervention, rehabilitation could be tailored to cognitive profiles. For example, participants with impaired executive function might be candidates for integrated cognitive-motor training to explore its potential to enhance postural control, while those with relatively preserved visual memory could be considered for a protocol with reduced visually guided training and increased eyes-closed balance exercises. The effects of such tailored approaches should be evaluated on an individual basis and in future controlled studies to avoid overgeneralization.

This study has several limitations. Its cross-sectional design precludes causal inferences, and longitudinal studies are needed to validate the predictive role of cognitive domains. Although the sample size was adequate for detecting medium effects, it may be under powered to identify weaker associations. Not all potential confounders were included, which may affect the precision of the estimates. As participants were primarily in the acute or subacute phases, generalizing findings to other stages or populations requires caution. Finally, multiple statistical tests were conducted across regression models without formal correction for multiple comparisons, and the analysis was restricted to linear associations. Consequently, the findings are preliminary and require confirmation in future studies with larger samples, comprehensive covariate control, and analytical methods capable of exploring non-linear relationships.

## Conclusion

This exploratory study indicates that specific cognitive domains, notably executive function and visual memory, are robustly associated with distinct aspects of postural control but demonstrate limited association with single-task functional walking measures. This pattern underscores the centrality of cognitive-motor task demand matching in understanding cognition-motor interactions.

## Data Availability

The datasets presented in this article are not readily available because they contain sensitive information that could compromise the privacy of the research participants. Access to de-identified datasets requires approval from the First Rehabilitation Hospital of Shanghai (YK20240308-01-006) Ethics Committee/Institutional Review Board (IRB) and a formal Data Use Agreement (DUA) to ensure compliance with ethical standards and data protection regulations (e.g., GDPR/HIPAA). Requests to access these datasets should be directed to the ethics committee of the First Rehabilitation Hospital of Shanghai. Requests to access the datasets should be directed to wanglu980114@163.com.
